# A new species of dragon’s blood *Croton* (Euphorbiaceae) from Serra dos Órgãos (Rio de Janeiro, Brazil)

**DOI:** 10.3897/phytokeys.126.35649

**Published:** 2019-06-28

**Authors:** Sabrina Queiroz de Farias, Débora Medeiros, Ricarda Riina

**Affiliations:** 1 Universidade Federal do Rio de Janeiro, Museu Nacional, Departamento de Botânica, Quinta da Boa Vista s/n, São Cristóvão, CEP: 20.940-040, Rio de Janeiro, RJ, Brazil Universidade Federal do Rio de Janeiro Rio de Janeiro Brazil; 2 Real Jardín Botánico, RJB-CSIC, Plaza de Murillo 2, 28014 Madrid, Spain Real Jardín Botánico Madrid Spain

**Keywords:** Atlantic Rain Forest, *Croton* sect. *Cyclostigma*, Neotropics, taxonomy

## Abstract

*Crotonrizzinii* Farias & Riina, **sp. nov.** is a new species from Serra dos Órgãos National Park in the Atlantic Rain Forest domain (Rio de Janeiro state, Brazil). It is known from the municipalities of Guapimirim, Teresópolis and Petrópolis, where it grows in montane ombrophilous dense forest, between 500 and 1500 m elevation. This arborescent species belongs to CrotonsectionCyclostigma Griseb., a Neotropical lineage distributed in forest habitats from Mexico to northern Argentina. It is mainly characterised by its laciniate-glandular stipules, bracts with two inconspicuous glands (colleters) at the base and campanulate pistillate flowers with sepals covering the ovary. We describe and illustrate the new species, and compare it with close relatives occurring in the Atlantic Rain Forest. We also provide a distribution map, habitat information and suggestions for the assessment of its conservation status.

## Abstract (es)

*Crotonrizzinii* Farias & Riina, **sp. nov.**, é uma nova espécie do Parque Nacional da Serra dos Órgãos, no domínio da Mata Atlântica (Rio de Janeiro, Brasil). É conhecida nos municípios de Guapimirim, Teresópolis e Petrópolis, onde cresce em floresta ombrófila densa montana, entre 500 e 1500 m de elevação. Esta espécie arborescente pertence a *Croton* seção *Cyclostigma* Griseb., uma linhagem de espécies neotropicais distribuídas em habitats florestais desde o México ao norte da Argentina. Caracteriza-se, principalmente, pelas estípulas laciniado-glandulares, brácteas com duas glândulas inconspícuas (coléteres) na base e flores pistiladas campanuladas com sépalas cobrindo o ovário. Descrevemos e ilustramos a nova espécie e a comparamos com espécies relacionadas ocorrentes na Mata Atlântica. Incluímos um mapa de distribuição, informações de habitat e recomendações para a avaliação do estado de conservação da espécie.

## Introduction

The Atlantic Rain Forest (ARF) is the richest Brazilian domain for flowering plants (BFG 2018) and one of the world hotspots of biodiversity (Myers et al. 2000). Not surprisingly, the megadiverse genus *Croton* L., with around 1200 spp. (Berry et al. 2005), is well represented in this region. Of the 305 species of *Croton* known for Brazil, 96 occur in the ARF (Flora do Brasil in prep), of which 21 species are trees (Santos et al. 2017). Amongst these arborescent lineages, C.sect.Cyclostigma is the most diverse in the ARF, having nine species (Santos et al. 2017).

While working on *Croton* for the Flora do Brasil project, we came across several specimens from Serra dos Órgãos that we could not place to species, all of which were collected within the limits of the eponymous national park (PARNASO). These specimens were from small trees and detailed examination of their morphology showed a clear affinity to species in sectionCyclostigma.

CrotonsectionCyclostigma, which includes species commonly known as dragon’s blood, comprises 46 species distributed from Mexico to northern Argentina (Feio et al. 2018a). The new species, described here, increases the total number in the section to 47 and to ten species in the ARF. This section is characterised by fast-growing trees, generally occurring in secondary vegetation, roadsides, river banks and landslide areas of wet forests (Riina et al. 2009), but there are a few species also occurring in dry forests (Riina et al. 2007, Feio et al. 2018a). The common name, dragon’s blood, comes from the presence of abundant and often reddish latex, which is used by indigenous and rural populations for wound-healing, preventing infections and other ailments (Ubillas et al. 1994, Jones 2003, Smith 2006).

The Serra dos Órgãos mountain range is part of the Serra do Mar mountain system in Rio de Janeiro state. The PARNASO was created in 1939 and it covers the municipalities of Guapimirim, Magé, Petrópolis and Teresópolis (Cronemberger and Castro 2007). Most of the park’s vegetation consists of dense ombrophilous forests, but there are also sparse areas with open vegetation or “campos de altitude” (Veloso et al. 1991). The great elevational gradient (ca. 100 to 2285 m) in a relatively small area has been suggested as the main factor in the formation of this unique and highly diverse environment (Cronemberger and Castro 2007).

Our study contributes to the botanical knowledge of the ARF region by describing a new tree species, *Crotonrizzinii*. We provide a complete morphological description, detailed illustrations and a distribution map. We also compare the new taxon with the morphologically most similar species in section Cyclostigma occurring in the ARF area.

## Materials and methods

We carried out fieldwork between 2015 and 2017 in different areas of the PARNASO, focusing on *Croton* species. Species were randomly sampled at each locality and the individuals were revisited during the following two years after the initial collections. The description of the new species was based on the analysis of dry plant material deposited in herbaria, including recently collected specimens, as well as field observations. The comparison with related species was undertaken using specimens from the herbaria R and RB, as well as photographs of specimens, including types, from herbaria F, G, K, NY, P, US and W (acronyms follow Index Herbariorum, available at https://sweetgum.nybg.org/science/ih/). We also used protologues of *Croton* species known from the ARF and recent floristic studies, particularly that of Santos et al. (2017). Morphological terms follow Hickey and King (2000) and Radford et al. (1974) for leaf morphology, Webster et al. (1996) and Feio et al. (2018b) for trichomes and Vitarelli et al. (2015) and Feio et al. (2016) for secretory structures. The classification of habitat (vegetation type) follows Veloso et al. (1991). The distribution map was produced using the software QGIS version 2.14 (QGIS Development Team 2019), based on geographic coordinates obtained during the collection expeditions and from herbarium specimens. Our suggestion for conservation status was based on field observations, IUCN Red List Categories and Criteria (IUCN 2017) and geospatial conservation assessment (GeoCAT) (Bachman et al. 2011). The extension of occurrence (EOO) was calculated using the GeoCAT online tool (http://geocat.kew.org).

## Results

### Taxonomic treatment

#### Croton rizzinii

Taxon classificationPlantaeMalpighialesEuphorbiaceae

Farias & Riina sp. nov.

urn:lsid:ipni.org:names:77199042-1

Figures 1 , 2

##### Diagnosis.

*Crotonrizzinii* is similar to *Crotonceltidifolius* Baill., but differs from the latter by the yellowish latex (vs. ochraceous to reddish latex), branches with appressed to stipitate stellate-porrect, stipitate-fasciculate and appressed-multiradiate trichomes (vs. appressed to stipitate stellate, stellate-porrect, appressed dendritic and dendritic-porrect trichomes), stipules (10–)12–14 mm long, laciniate-glandular (vs. 2–6 mm long, entire, eglandular), bracts with two inconspicuous glands (colleters) at the base (vs. eglandular bracts), staminate flowers with 64–76 stamens (vs. 50–60), campanulate pistillate flowers, sepals ca. 6–9.5 mm long, valvate to slightly imbricate (vs. rotate, ca. 2.5–5 mm long, valvate).

##### Type.

BRAZIL. Rio de Janeiro: Teresópolis, Parque Nacional da Serra dos Órgãos, BR-495, rodovia Teresópolis-Itaipava, em borda de mata de encosta, 22°24'40.8"S, 43°02'08.5"W, alt. 1414 m, 28 Jan 2017, fl, S.Q. Farias & J.L. Silva 205 (holotype: R!; isotype: RB!).

##### Description.

***Trees*** ca. 5–10 m tall, monoecious; young branches with a dense to sparse indumentum of appressed to stipitate stellate-porrect, stipitate-fasciculate and appressed-multiradiate trichomes, cinereous, yellowish, or pale ferrugineous; latex yellowish. ***Leaves*** alternate, simple; lamina 7–22 × 2.4–12.2 cm, discolorous, membranaceous, ovate to cordate, apex acute, long-acuminate to caudate-acuminate, base rounded, obtuse to cordate, adaxial surface sparsely pubescent with indumentum of appressed to short-stipitate fasciculate trichomes, more concentrated along the veins, abaxial surface densely pubescent with indumentum of stipitate stellate-porrect to stipitate-multiradiate trichomes; venation brochidodromous, trinerved at the base to palminerved; margin inconspicuously dentate, with ovoid to cylindrical glands (colleters) at the tip of each tooth; petioles 1.9–13 cm long, with dense indumentum of appressed to stipitate stellate-porrect, stipitate-fasciculate and appressed-multiradiate trichomes; nectary glands 2, acropetiolar, patelliform, shortly stipitate to stipitate, on the abaxial side; stipules (10–)12–14 mm long, linear-lanceolate, cream to yellowish-green, margin laciniate-glandular, sometimes with a glandular tip, abaxial surface with sparse indumentum of appressed stellate-porrect to multiradiate trichomes. ***Inflorescences*** terminal, erect, 6–29 cm long, axis with dense indumentum of appressed to stipitate stellate-porrect trichomes; proximal cymules bisexual, spaced along the axis; cymule bracts variable in size and shape, 4–9 × 1.6–2 mm, narrowly oblong, linear-lanceolate or lanceolate, abaxial surface with appressed stellate-porrect trichomes, with 2 sessile, minute, basal glands (colleters), margin entire to irregularly dissected with stipitate-fasciculate trichomes. ***Staminate flowers*** rotate, 7–11 mm long; pedicel 2.5–6 mm long, with indumentum of appressed stellate-porrect trichomes; sepals 5, valvate, connate at the base, entire, equal, 3.5–4 × 2–2.5 mm, ovate, apex acute, adaxial surface with simple trichomes at the base and along the margin; abaxial surface with appressed stellate-porrect trichomes; petals 5, 2.2–3.5 × 0.9–1.2 mm, distinct, oblanceolate, apex acute, adaxial surface with simple trichomes at the base, abaxial surface with simple trichomes along the margin; stamens 64–76, filaments 2.5–4.5 mm long, with simple trichomes, disc 5-segmented, receptacle villose with dense simple trichomes. ***Pistillate flowers*** campanulate, 6.5–10.1 mm long, sessile to subsessile; sepals 5, 6–9.5 × 4–5 mm, valvate to slightly imbricate, connate at the base, entire, unequal, ovate to broadly ovate, apex acute to abruptly acute with glandular tip (colleter), adaxial surface with indumentum of short-stipitate stellate trichomes at the distal portion, abaxial surface with indumentum of appressed stellate-porrect and appressed-multiradiate trichomes; maculate glands sometimes present on some of the sepals; petals each reduced to a filament with an apical gland (colleter); ovary ca. 3.4 mm in diam., densely covered with pale ferrugineous rosulate trichomes; styles 3, 4-fid to multifid (12–18 terminal tips), connate at the base, with appressed stellate-porrect trichomes; disc 5-segmented. ***Capsules*** 7.5–10 × 4.8–5.4 mm, subglobose, densely covered with pale ferrugineous rosulate trichomes; sepals and columella persistent; columella 6 mm long. ***Seeds*** 4.1–5.4 × 3–3.3 mm, oblongoid, brown, ribbed; caruncle 1 × 2.5 mm, cream, transversely oblong.

**Figure 1.**
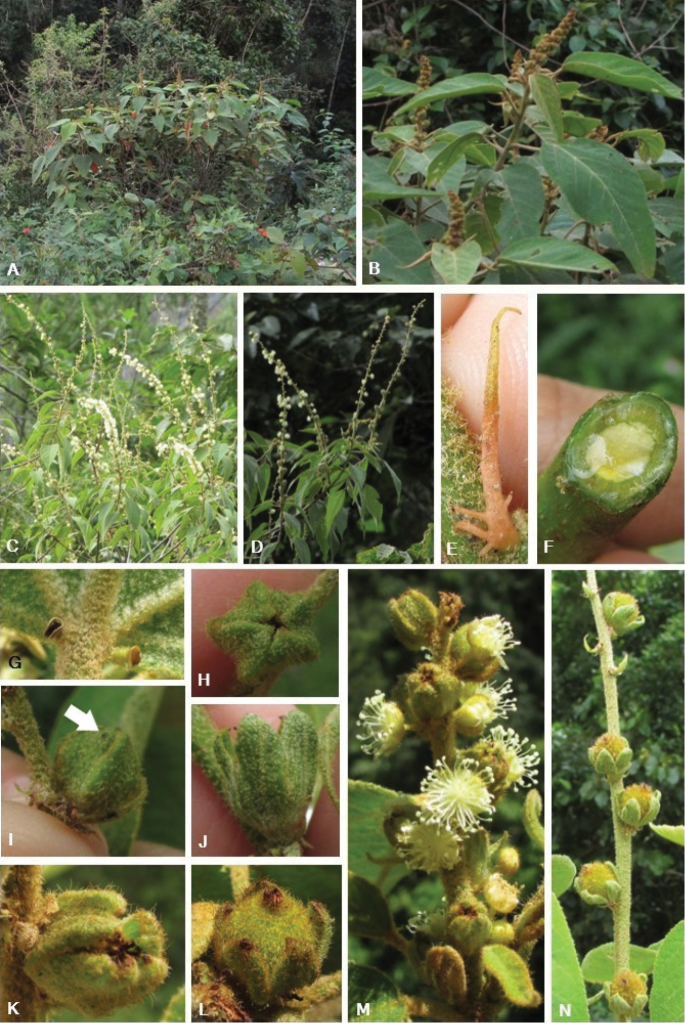
*Crotonrizzinii*. **A** Adult individual in hillside forest **B** young flowering branch **C** mature flowering branch **D** detail of a mature flowering branch **E** detail of laciniate-glandular stipules **F** young branch showing yellowish latex **G** detail of leaf showing acropetiolar glands **H–K** stages of development of pistillate flowers **H** top view of a young pistillate flower **I** young pistillate flower showing a maculate gland on the distal portion of the sepal **J** young pistillate flower **K** mature pistillate flower **L** fruit **M** inflorescence showing pistillate and staminate flowers **N** inflorescence showing fruits. (Photos by S.Q. Farias).

**Figure 2.**
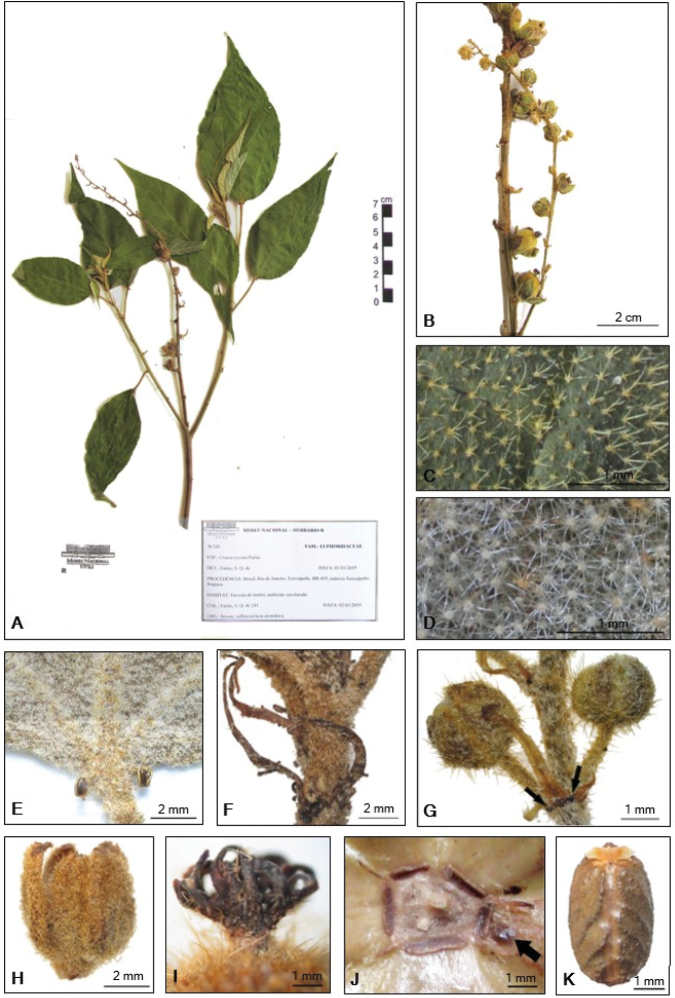
Images from herbarium specimens of *Crotonrizzinii*. **A** Flowering branch **B** inflorescence **C** indumentum on the adaxial surface of the lamina **D** indumentum on the abaxial surface of the lamina **E** detail of leaf showing the two acropetiolar glands **F** stipules **G** detail of a bract of a cymule with glands (colleters) at the base **H** mature pistillate flower **I** detail of the styles connate at the base **J** pistillate flower with ovary removed, showing disc and a gland at the base of one of the sepals **K** seed (ventral surface). (**A, B***S.Q*. *Farias 241***C, D, G***S.Q. Farias & J.L. Silva 239***E, F, K***S.Q. Farias & J.L. Silva 234*; **H, I, J***S.Q. Farias & J.L. Silva 205*, holotype).

##### Specimens examined.

BRAZIL – Rio de Janeiro • A. Souza et al. 1576 (R); Teresópolis [Guapimirim], Serra dos Órgãos, rodovia Rio-Teresópolis, próximo ao rio Soberbo; 22°29'23.56"S, 43°00'27.71"W; alt. 505 m; 28 Nov. 1986; fl • A. Souza et al. 1575 (R); Petrópolis, Parque Nacional da Serra dos Órgãos, BR-495, rodovia Teresópolis-Itaipava; 28 Nov. 1986; fl • S.Q. Farias & J.L. Silva 239 (R); ibid, km 9; 22°24'38.5"S, 43°02'24.2"W; alt. 1416 m; 28 Apr. 2018; fl • R. Barnes s.n. (R 186364, FUEL34028, INPA 212592, IPA 61764, MBM 275097, RB 377649, SP 361735); Teresópolis; 30 Jun. 1995; fl • R. Barnes s.n. (R 185344); Serra dos Órgãos, próximo ao abrigo 3; 22°26'54.32"S, 42°59'0.21"W; alt. 1000 m; 25 Jan. 1995; fl • S.Q. Farias & J.L. Silva 200 (R); BR-495, rodovia Teresópolis-Itaipava; 22°24'40.8"S, 43°02'08.5"W; alt. 1414 m; 15 Jan. 2017; fl, fr • S.Q. Farias & J.L. Silva 234 (R, RB); ibid; 2 Apr. 2018; fl, fr • S.Q. Farias 241 (R, RB); ibid; 2 Mar. 2019; fl, fr.

##### Distribution and habitat.

**Figure 3.**
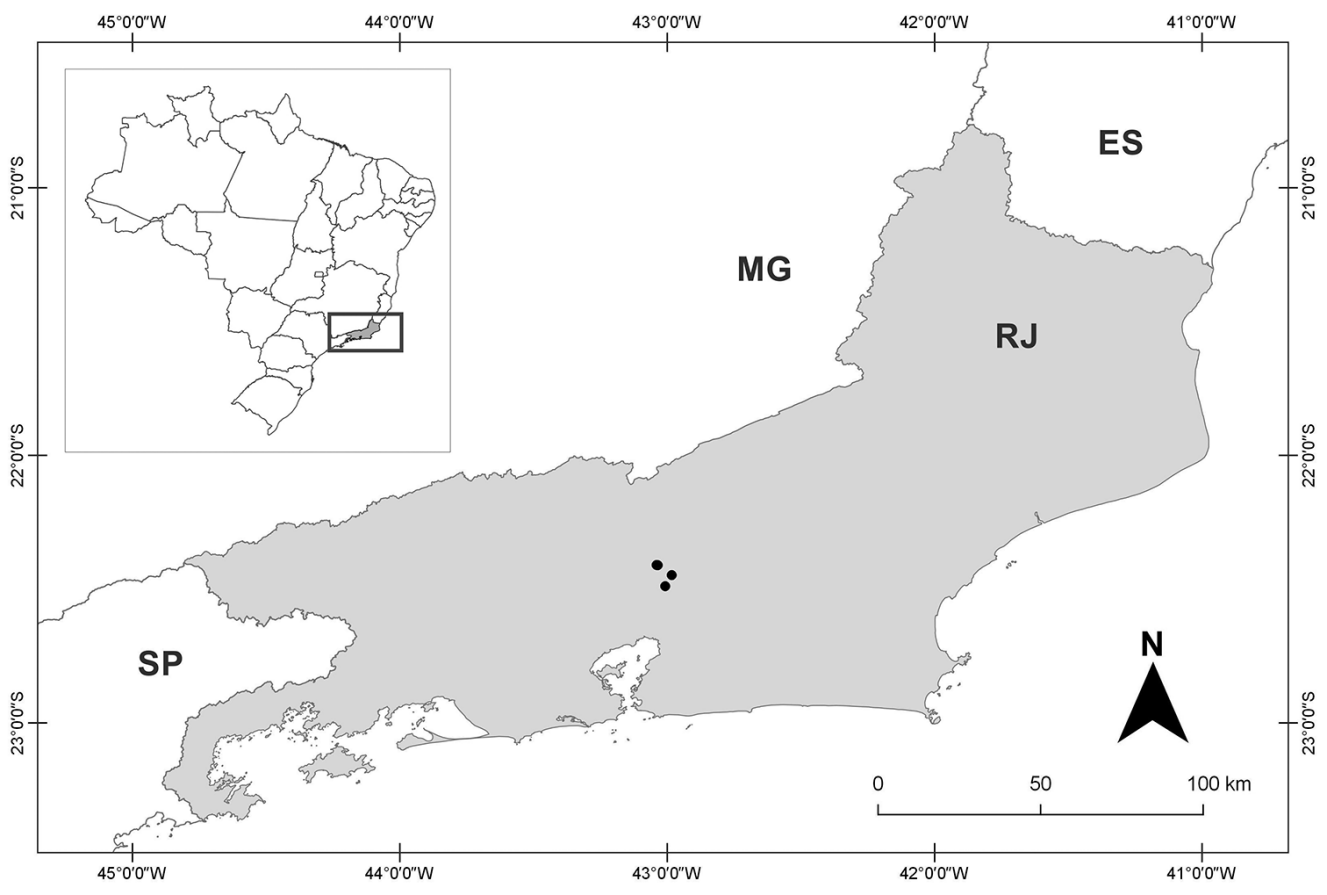
Map of Rio de Janeiro State showing the distribution of *Crotonrizzinii* (black circles). The inset map of Brazil on the left provides the context with the state of Rio de Janeiro highlighted in grey. ES = Espírito Santo, MG = Minas Gerais, RJ = Rio de Janeiro, SP = São Paulo.

*Crotonrizzinii* is only known from the Serra dos Órgãos National Park, with records in the municipalities of Guapimirim, Petrópolis and Teresópolis (Rio de Janeiro) (Figure 3). It grows in montane ombrophilous dense forest, between 500 and 1500 m elevation and in disturbed areas like forest edges and roadsides.

##### Phenology.

The new species has been collected in flower in January, March, April, June, November and December. The flowering peak is in December and January, with fruits between January and April.

##### Etymology.

The specific epithet is given in homage to Carlos Toledo Rizzini, a Brazilian botanist, for his dedication to the study of the flora of the Serra dos Órgãos mountain range.

##### Conservation status.

*Crotonrizzinii* is known from a few collections in three municipalities of the PARNASO. It has an estimated Extent of Occurrence (EOO) of 19,653 km^2^. Although the species occurs within a conservation unit, it suffers considerably from anthropic pressure caused by tourism, urbanisation, agriculture and grazing. Based on our data, *Crotonrizzinii* could be assessed either as Critically Endangered (CR B1b[iii]) or Data Deficient (DD). The latter category is based on the lack of sufficient information regarding the distribution and size of populations.

##### Discussion.

*Crotonrizzinii* can be recognised in the field by its yellowish latex, laciniate-glandular stipules that are cream to green-yellowish in colour, bracts with two inconspicuous glands (colleters) at the base, campanulate pistillate flowers with sepals covering the entire ovary and the presence of maculate glands on the adaxial surface. We are assigning the species to Crotonsect.Cyclostigma (Van Ee et al. 2011) due to its arborescent habit, yellowish latex, stellate trichomes, triplinerved to palmate venation, acropetiolar glands, basal bisexual cymules, pistillate flowers with vestigial petals and staminate flowers with numerous stamens. However, further testing using molecular characters would be desirable to confirm its phylogenetic position. The finding of a new species in this section in a relatively well explored area is surprising and shows that botanical exploration continues to be relevant in highly diverse Neotropical areas and especially in biodiversity hotspots (Myers et al. 2000, Joppa et al. 2011) regardless of their current level of floristic inventory and the taxonomic knowledge of the focal group (Riina et al. 2018).

*Crotonrizzinii* can be distinguished from other Brazilian *Cyclostigma* species by its laciniate-glandular stipules, bracts with glands (colleters) at the base, sepals of the pistillate flowers with glands (colleters) at the apex and, sometimes, macular glands on the adaxial surface. Another character distinguishing it from its most similar species in the ARF is the size of seeds, which are the smallest in size amongst them (Table 1). Amongst the ARF*Cyclostigma* species, *Crotonrizzinii* appears to be most similar to *C.celtidifolius* (Santos et al. 2017; Caruzo and Cordeiro 2007). Both species occur sympatrically and syntopically and can be confused by their similar ovate to cordate and pubescent leaves, trinerved at the base to palminerved venation, short-stipitate to stipitate petiolar glands on the abaxial side of the petiole and subglobose fruits. However, they can be readily distinguished by several characters, mainly by those related to latex colour, type of trichomes on branches, number of acropetiolar glands, stipules, bracteoles, stamen number and pistillate flowers (see Table 1 and identification key). Due to their resemblance, specimens of *C.rizzinii* have often misidentified as *C.celtidifolius* in herbaria.

**Table 1.** Main characters distinguishing *Crotonrizzinii* from its morphologically closest species in the Atlantic Rain Forest (ARF).

**Characters**	*** C. rizzinii ***	*** C. alchorneicarpus ***	*** C. celtidifolius ***	*** C. lagoensis ***	*** C. vulnerarius ***
Maximum height (m)	10	15	15	4	10
Trichomes on branches	Appressed to stipitate stellate-porrect, stipitate-fasciculate and appressed-multiradiate	Appressed stellate, stellate-porrect, dendritic and dendritic-porrect	Appressed to stipitate stellate, stellate-porrect, appressed dendritic and dendritic-porrect	Appressed to stipitate stellate-lepidote	Appressed-stellate, stipitate-dendritic and appressed-rosulate
Petiolar glands	2, short-stipitate to stipitate	2(–4), stipitate	2(–4), stipitate, rarely short-stipitate	2, sessile	2(–4), stipitate
Stipule shape	Linear-lanceolate, never foliaceous	Linear to lanceolate, sometimes foliaceous	Linear to lanceolate, rarely foliaceous	Linear-lanceolate to lanceolate, never foliaceous	Lanceolate to filiform, never foliaceous
Stipule margin	Laciniate-glandular	Entire	Entire	Entire	Entire
Stipule length (mm)	(10–)12–14	6–12	2–6	6–8	(9–)13–16
Inflorescence length (cm)	6–29	6–13	(11–)22–32	10–15	7–12
Bracts of the cymules	Entire to irregularly dissected, 2 glands at the base	Entire, eglandular	Entire, eglandular	Entire, eglandular	Entire, eglandular
Staminate flower shape	Rotate	Rotate	Rotate	Rotate	Subcampanulate
Stamen number	64–76	ca. 60	50–60	40–50	ca. 100
Pistillate flower shape	Campanulate	Subcampanulate	Rotate	Campanulate	Campanulate
Pistillate sepal shape	Ovate to broadly ovate	Broadly ovate	Ovate to lanceolate	Broadly ovate	Ovate-lanceolate
Pistillate sepal length (mm)	6–9.5	5–9.4	2.5–5	3–6	5.5–7.5
Vestigial pistillate petals	Filiform	Filiform	Filiform	Filiform	Linear-lanceolate
Seed size (mm)	4.1–5.4 × 3–3.3	7.5 × 4	5.7–7 × 3.7–4	7 × 4	5.9 × 3.4
Habitat	Montane wet forest	Montane wet forest, riparian forest	Montane wet forest, dry forest riparian forest	Dry forest, campos rupestres	Montane wet forest, dry forest
Elevation (m)	500–1500	800–2320	350–1400	800–1800	780–1600
Distribution	ARF (RJ)	ARF (MG, SP, RJ)	ARF (ES, MG, PR, RJ, SC, SP, RS)	Between ARF and Cerrado (MG)	ARF (MG, SP, RJ, PR)

*Crotonrizzinii* is also similar to *C.vulnerarius* Baill. and *C.alchorneicarpus* Croizat, mostly regarding the young pistillate flowers. *Crotonvulnerarius* and *C.rizzinii* occur sympatrically within PARNASO, but so far, they have not been found in the same locality. These species share an arborescent habit, pubescent leaves, conspicuous stipules, sessile to subsessile campanulate pistillate flowers, valvate to slightly imbricate sepals and styles connate at the base. Nevertheless, they can be distinguished by several characters (see Table 1 and identification key). In relation to *C.alchorneicarpus*, both species present ovate to cordate leaves, trinerved at the base to palminerved venation, conspicuous stipules, sessile to subsessile pistillate flowers with styles connate at the base, rotate staminate flowers and subglobose fruits. However, they too can be separated by several vegetative and reproductive characters (see Table 1 and identification key).

Several collections (Cordeiro 3056, 3057, Riina and Caruzo 1526, 1529) from ARF areas of São Paulo and Rio de Janeiro states show characteristics intermediate between *Crotonalchorneicarpus* and *C.rizzinii.* The overall aspect of the plant and the floral morphology are more similar to *C.alchorneicarpus*, but the stipules are more similar to those of *C.rizzinii* (laciniate-glandular). Further studies and additional sampling of *Croton* trees from the ARF are needed to determine the identity of these specimens.

*Crotonlagoensis* Müll. Arg. is also similar to *C.rizzinii*, but it occurs in deciduous forest and in transitional areas between the Cerrado and the ARF in the state of Minas Gerais (Santos et al. 2017). Both species have ovate to cordate and pubescent leaves, two acropetiolar glands and campanulate pistillate flowers with sepals covering the entire ovary. However, *C.rizzinii* differs from *C.lagoensis* mainly by its arborescent habit and other features listed in Table 1 and in the identification key.

The description of *C.rizzinii* adds to the number of species with laciniate stipules in *Croton* and in section Cyclostigma in particular. Laciniate stipules are found in seven species in *Cyclostigma* (*C.charaguensis* Standl., *C.churutensis* Riina & Cornejo, *C.medusae* Müll. Arg., *C.perspeciosus* Croizat, *C.purdiei* Müll. Arg., *C.rizzinii* and *C.speciosus* Müll. Arg.), but they can be present in other sections of Croton (sect. AdenophylliGriseb., sect. Barhamia (Klotzsch) Baill., sect. Medea (Klotzsch) Pax, amongst others) (Van Ee et al. 2011). However, given the inconsistencies found in *Croton* taxonomic treatments regarding the terms used to describe stipules, we suggest further studies to standardise this terminology across the genus.

### Identification key for *Crotonrizzinii* and morphologically similar species in the ARF

**Table:** 

1	Habit shrubby; acropetiolar glands sessile	*** C. lagoensis ***
–	Habit arborescent; acropetiolar glands short-stipitate to stipitate	**2**
2	Latex yellowish; stipules laciniate-glandular; bracts with tiny glands (colleters) at the base; pistillate flowers campanulate, sepals with a tiny gland (colleter) at the apex	*** C. rizzinii ***
–	Latex translucid, ochraceous to reddish; stipules entire; bracts eglandular; pistillate flowers rotate, subcampanulate to campanulate, apex of sepals eglandular	**3**
3	Lamina pubescent on adaxial surface	**4**
–	Lamina glabrous to glabrescent on adaxial surface	*** C. alchorneicarpus ***
4	Stipules linear to lanceolate, 2–6 mm long; stamens 50–60; pistillate flowers rotate, pedicellate, rarely sessile; vestigial pistillate petals filiform	*** C. celtidifolius ***
–	Stipules lanceolate to filiform, (9–)13–16 mm long; stamens ca. 100; pistillate flowers campanulate, sessile to subsessile; vestigial pistillate petals linear-lanceolate	*** C. vulnerarius ***

## Supplementary Material

XML Treatment for Croton rizzinii
